# Spray-Dried Whey Protein Concentrate-Iron Complex: Preparation and Physicochemical Characterization

**DOI:** 10.17113/ftb.57.03.19.6228

**Published:** 2019-09

**Authors:** Indrajeet Singh Banjare, Kamal Gandhi, Khushbu Sao, Rajan Sharma

**Affiliations:** Dairy Chemistry Division, National Dairy Research Institute, P.O. Box 132001, Karnal, India

**Keywords:** fortification, whey protein concentrate, iron, spray drying, stability, bioaccessibility

## Abstract

Poor absorption of iron from food and oral iron formulations results in extensive use of high-dose oral iron, which is not tolerated. Disposal of whey, a byproduct of the cheese industry, causes environmental pollution. Whey proteins have the ability to bind significant amount of iron, thereby reducing its chemical reactivity and incompatibility with other components in foods. To make iron compatible with food, it was complexed with whey protein concentrate (WPC). After complexation, centrifugation and ultrafiltration techniques were utilised to eliminate the insoluble and free iron from the solution. To enable the availability of whey protein concentrate–iron (WPC–Fe) complex in the powder form, spray drying technique was used. Optimized spray drying conditions used for the preparation were: inlet temperature 180 °C, flow rate 2.66 mL/min and solution of total solids 15%. The complex was observed to be stable under different processing conditions. The *in vitro* bioaccessibility (iron uptake) of the bound iron from the WPC–Fe complex was significantly higher (p<0.05) than that from iron(II) sulphate under simulated gastrointestinal conditions. WPC–Fe complex with improved iron bioaccessibility could safely substitute iron fortificants in different functional food preparations.

## INTRODUCTION

Occurrence of anaemia in India is a major problem affecting foremost the pregnant women and preschool children (*1*). Factors responsible for the high incidence include inadequate dietary intake of iron, faulty iron absorption, increased iron demand during pregnancy and lactation, low iron reserves at birth, timing of umbilical cord clamping, occurrence of illnesses in children and excessive loss of blood during puberty and pregnancy (*2*). Food fortification with iron has been recommended as one of the favoured approaches for averting and eliminating the iron deficiency. However, fortification of foods with iron salts results in metallic aftertaste, undesirable flavour due to the oxidation-mediated rancidity of fats, undesirable colour changes as a result of interactions with flavonoids, anthocyanins and tannins, and degradation of vitamins and minerals (*3*). Whey, a liquid byproduct of cheese and casein industry, is one of the good sources of protein but remains unutilised for the consumption.

Whey proteins have higher nutritional value and better functional properties than casein and other food proteins, which promotes their wide use as food constituents such as whey protein isolate or whey protein concentrate (WPC) (*4*). WPC, an industrially available protein, because of its structural and functional properties, was used in this study for the complex formation with the iron in order to make it compatible with the food products. Moreover, it acts as a natural antioxidant, adding to the stability of the complex. Added iron primarily binds with oxygen atoms of phosphoserine, aspartic and glutamic acid residues *via* coordination bonds on individual proteins (*5*). There are only a few studies performed so far on the binding mechanism of iron to WPC followed by its application in milk and milk products. Centrifugation and ultrafiltration techniques were used to remove the insoluble and free iron, respectively, while the spray drying was used to obtain WPC–Fe complex in powder form. Physicochemical properties and stability of the formed complex were evaluated under different processing conditions. In a previous study (*6*), lyophilisation was used to obtain WPC–Fe complex in powder form, but since it is a time consuming and costly process (*7*), it cannot be scaled up to the industrial level. Further, to produce the particles of definite size, it requires an additional milling step (*8*).

Spray drying is a major technique for the stabilisation of the milk constituents and production of milk powders (*9*). It is preferred in food processing to conventional drying methods (*10*), as it is fast, relatively cheap and highly reproducible (*11*). Unlike lyophilisation, it is a ‘continuous process’ and can be used when bulk production of the powder is required. Incorporating iron in dairy ingredients like WPC could deliver it to all age groups and sections of the society. Therefore, laboratory scale spray dryer was used for the production of WPC–Fe complex, followed by its physicochemical characterization.

## MATERIALS AND METHODS

### Materials

Whey protein concentrate (WPC 80) and iron(II) sulphate heptahydrate (FeSO_4_·7H_2_O) were obtained from Davisco Foods International Co. (Le Suer, MN, USA) and Sigma-Aldrich, Merck (St. Louis, MO, USA), respectively.

### Preparation of WPC–Fe complex

WPC–Fe complex was prepared by following the method of Banjare *et al.* (*8*) and Gandhi *et al.* (*12*). Iron solution was added slowly to 1.0% WPC solution prepared in deionised water with constant stirring at 600 rpm using magnetic stirrer (SPINOT MC 02; Tarsons Products Pvt. Ltd., Kolkata, India) to obtain final concentration of 3 mM. After adjusting the pH to 6.6, the solution was kept at 20 °C for 2 h. The solution was then centrifuged (high-speed refrigerated centrifuge model 6500; KUBOTA Corporation, Tokyo, Japan) at 12 000×*g* and 20 °C for 30 min. Soluble iron and protein present in the supernatant were carefully decanted and filtered through Whatman No. 1 filter paper (Whatman International Ltd., Kent, UK). To separate the soluble bound (retentate) from the soluble free iron (permeate), the filtered supernatant was then passed through a Hydrosart ultrafiltration cassette (*M*=10 kDa; Sartorius India Pvt. Ltd., Mumbai, India). The obtained retentate was concentrated to 15% total solids (TS) and then spray dried.

### Spray drying of WPC–Fe complex solution

Spray-dried WPC–Fe complex was prepared using a table top spray dryer (SPD-P-111; Technosearch Instruments, Mumbai, India) equipped with a co-current nozzle. The processing conditions were: inlet air temperature 180 °C, TS of the WPC–Fe complex solution 15% and flow rate 2.66 mL/min optimized with central composite rotatable design (CCRD) of response surface methodology using Design-Expert v. 10.0.8 (*13*) software to obtain maximum yield and solubility and minimum moisture content.

### Moisture content

Moisture content was determined using the gravimetric method (*14*). Approximately 1.0 g of sample was weighed in a moisture dish covered with a lid and the mass was recorded. The dish was then uncovered and placed in the oven (Tempo Instruments Pvt. Ltd., Mumbai, India) at (102±2) °C for 2 h along with the lid. After drying, the dish was again covered with the lid and transferred to the desiccator to cool to room temperature. Then, it was carefully weighed and the process was repeated until the mass did not differ by more than 0.5 mg between measurements. Moisture content was then calculated from the following formula:


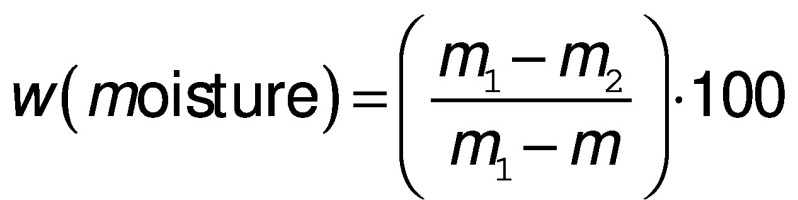


where *m* is the mass of the empty dish (g), *m*_1_ is the initial mass of the dish containing the sample (g) along with the lid, and *m*_2_ is the final mass of the dish with the sample and lid after drying (g).

### Protein content

The protein content was calculated with the following formula:

*w*(protein)=*w*(N)·6.38 /2/

Nitrogen in each sample was determined according to the AOAC official method 981.10 (*15*). A mass of 100 mg of the sample was weighed into 100-mL Kjeldahl flask and 20 mL concentrated sulfuric acid (Thermo Fisher Scientific, Mumbai, India) were added. Copper sulphate 0.2 g and potassium sulphate 10 g (both Sigma-Aldrich, Merck) were then added. The content was digested with the digestion mixture until the clear solution was obtained. After cooling, the content was transferred to a 100-mL volumetric flask and the volume was made up to the mark. Diluted sample (10 mL) was then transferred into Kjeldahl flask. Tip of the condenser was dipped in a flask containing 10 mL saturated boric acid solution (Sigma-Aldrich, Merck) containing a mixed indicator (methyl red solution and methylene blue solution (both from HiMedia Laboratories Pvt. Ltd., Mumbai, India) in the ratio 1:1.

A volume of 20 mL of 50% NaOH (Sigma-Aldrich, Merck) and a small amount of distilled water were then added. Distillation continued until about 50 mL of the distillate were collected in the conical flask. The content of the conical flask was then titrated with 0.02 M HCl (Thermo Fisher Scientific). A blank determination was also carried out using sucrose (Sigma-Aldrich, Merck) instead of the sample. Nitrogen content expressed as percentage was calculated using the following formula:


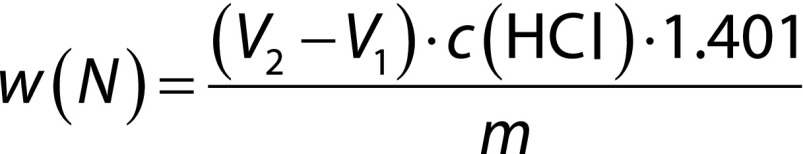


where *V*_2_ is the amount of acid required for sample titration (mL), *V*_1_ is the amount of acid required for blank titration (mL), *c*(HCl) is molar concentration of HCl used for titration (0.02 M), 1.401 is the molar mass of nitrogen and *m* is the mass of sample (g).

### Lactose content

Lactose content was determined using the photometric method (*16*). A mass of 0.5 g of sample was dissolved in distilled water and the volume was made up to 100 mL in a volumetric flask. A volume of 0.4 mL of the sample was pipetted into a test tube and 0.4 mL phenol solution (Sigma-Aldrich, Merck) was then added with proper mixing. A volume of 2 mL of concentrated sulfuric acid (Thermo Fisher Scientific) was then added and mixed immediately using vortex mixer (Spinix vortex shaker 3020; Tarsons Products Pvt. Ltd) for 30 s. After 10 min, when the temperature reached 30 °C, the absorbance of the solution was measured at 490 nm using the blank solution as reference. Reagents were added to the blank solution containing 0.4 mL of distilled water instead of the sample. Calibration curve was constructed by plotting the absorbance against the concentration of lactose in µg/mL and the mass fraction of lactose in the sample was calculated from the following formula:


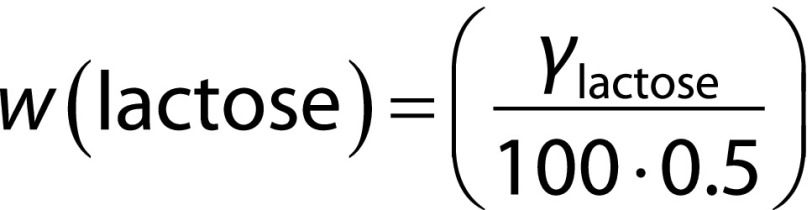


where *γ*_lactose_ is the concentration (µg/mL) of anhydrous lactose in the analysed solution as read from the calibration curve and 0.5 is the amount of the sample taken.

### Fat content

Fat content was determined by using the gravimetric method (*17*). A mass of 2 g of the sample was transferred into Mojonnier flask. Hydrochloric acid solution (Thermo Fisher Scientific) (10 mL) was then added into the bulb of the extraction flask. Mojonnier flask was gently heated in a boiling water bath (SUB Aqua 18 Plus; Grant Instruments, Cambridge, UK) to avoid charring until all the particles were dissolved completely. The flask was left to stand for 20-30 min in boiling water bath and then cooled under running tap water. A volume of 10 mL of ethanol (Sigma-Aldrich, Merck) was then added and mixed gently but thoroughly to allow the content of the flask to flow forwards and backwards between the bulb and the body of the flask. Diethyl ether (25 mL; Sigma-Aldrich, Merck) was then added, the flask was closed with a cork dipped in water at 60-70 °C for 10-15 min and the content mixed properly. Petroleum ether (25 mL; Sigma-Aldrich, Merck) was then added and the previous step was repeated. The content was then allowed to stand for 30–45 min at room temperature (30 °C) until the upper clear layer got separated. The flask was then carefully decanted into the preweighed aluminium dish. The solvent was then evaporated by keeping the aluminium dish over the boiling water bath. The second extraction was then performed in the similar way using 5 mL ethanol and 25 mL of each petroleum and diethyl ether. The third extraction was carried out as described above but using 15 mL of diethyl ether and 15 mL of petroleum ether. After extraction, the solvent in the aluminium dish was evaporated in hot air oven (Tempo Instruments Pvt. Ltd.) at (102±2) °C for 1 h. After incubation, the dish containing fat was removed from the oven, cooled and weighed. Blank test was carried out simultaneously as described above for the sample, but using 10 mL of water instead of the sample. Fat content was calculated using the formula:


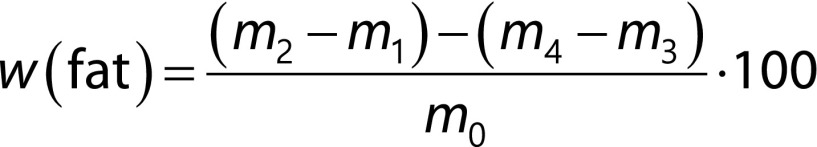


where *m*_0_ is the mass of the sample, *m*_1_ is the mass of empty sample aluminium dish, *m*_2_ is the mass of the aluminium dish with the extracted fat, *m*_3_ is the mass of the empty blank aluminium dish, and *m*_4_ is the mass of blank aluminium dish containing solvent extract.

### Ash content

Ash content was determined by using the IDF 90:2008(E) (*18*) method. A sample of 3 g was weighed into a previously dried silica crucible and weighed. The dish was gently heated on the flame and then ashed at 550 °C in a muffle furnace (Narang Scientific Works Pvt. Ltd., Delhi, India). The crucible was cooled in a desiccator, weighed, then heated again for 30 min in the muffle furnace and cooled. This heating and cooling process was repeated until the constant mass was obtained. Ash content was determined using the formula:


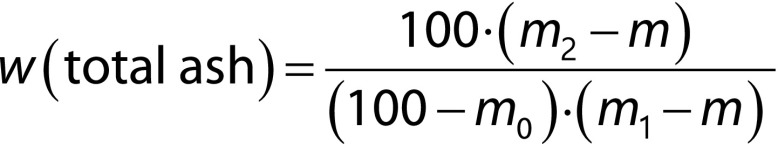


where *m* is the mass of the empty dish (g), *m*_1_ is the mass of the dish with the sample (g), *m*_2_ is the mass of the dish with the ash (g), and *m*_0_ is the moisture mass (%).

### Solubility

Solubility of WPC–Fe complex was determined by following the method of Elez Garofulić *et al.* (*19*) with modifications in the speed of centrifugation. Powder (1 g) was placed in a glass centrifuge tube with 10 mL distilled water and stirred vigorously for 1 min on a vortex vibrator (Spinix vortex shaker 3020; Tarsons Products Pvt. Ltd.), then kept in a water bath (SUB Aqua 18 Plus; Grant Instruments) at 37 °C for 30 min and finally centrifuged (R-8C; REMI Laboratory Instruments, Mumbai, India) at 1000 rpm for 10 min. The obtained supernatant was dried in a laboratory oven (Tempo Instruments Pvt. Ltd.) at 100 °C until a constant mass was obtained. Solubility was calculated according to the following formula:

Solubility=(*m*_s_/*m*_p_)·100 /7/

where *m*_s_ is the mass (g) of the supernatant obtained by drying, and *m*_p_ is the initial mass (g) of the powder used for analysis.

### Particle size and zeta potential

Dynamic light scattering and laser doppler microelectrophoresis technique were used to determine the particle size and zeta potential, respectively, of the WPC–Fe complex at 25 °C. Solution (1.0%, *m*/*V*) of the WPC–Fe complex was prepared in 0.05 M phosphate buffer (pH=7) and analysed using Zetasizer Nano ZS (Malvern Instruments Ltd., Malvern, UK).

### Stability of WPC–Fe complex

The impact of various processing steps on the stability (in terms of free iron release in permeate after ultrafiltration) was evaluated using the procedure described by Shilpashree *et al.* (*6*). The WPC–Fe complex solution (1%) was prepared in phosphate buffer (pH=7) and then the influence of pH (3, 4, 5, 6 and 7), heat (50, 60, 70, 80 and 90 °C/30 min) and ion concentration (0.1 to 0.5 M NaCl) was determined. Further, the solution was incubated at 30 °C for 24 h, followed by centrifugation (high-speed refrigerated centrifuge model 6500; KUBOTA Corporation) at 5000 rpm for 30 min. The stability expressed in terms of released free iron in permeate after ultrafiltration was analysed and calculated as follows:


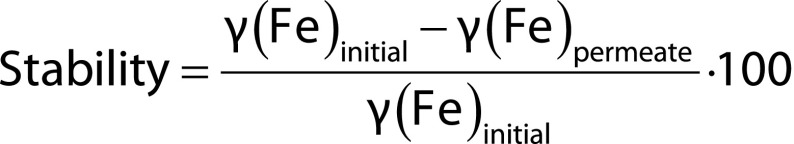


where *γ*(Fe)_initial_ is the initial iron concentration in the complex, and *γ*(Fe)_permeate_ is the iron concentration in the permeate after processing.

### Bulk density

Bulk density of the WPC–Fe complex was determined with ISO 8967/IDF 134:2005 method (*20*). Loose bulk density was determined in a 100-mL graduated cylinder, where dry powder was allowed to flow freely up to the 100 mL mark. The net mass of 100 mL powder was calculated and the result was expressed in g/mL. After calculating the loose bulk density, tapped bulk density was determined by manually tapping the cylinder about 50 times from a height of 10 cm on a solid marble surface to evaluate the final volume until a steady volume was obtained. Loose bulk density (*ρ*_B_) and tapped bulk density (*ρ*_T_) were calculated using the formulae:


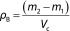


and


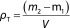


where *m*_1_ is the mass of graduated cylinder, *m*_2_ is the mass of cylinder containing WPC–Fe complex, *V*_c_ is the volume of cylinder (100 mL) and *V* is the volume after tapping.

### Flow properties

Carr index and Hausner ratio (*21*) served for the determination of flow characteristics of the WPC–Fe complex. The former measures the compressibility or free-flowing property, while the latter measures the powder's cohesiveness. The Carr index (C) and Hausner ratio (HR) were calculated using loose and tapped bulk density formulae as follows:


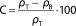


and


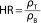


### Dissolution behaviour

The dissolution behaviour was measured spectrophotometrically (SPECORD®; Analytik Jena AG, Jena, Germany) as per the method of Millqvist-Fureby *et al.* (*22*). Powder sample (30 mg) was layered on top of 3 mL water in a cuvette (5×1×1 cm^3^) and the increase in the absorbance at 620 nm was recorded every minute until a constant reading indicating full dissolution was achieved.

### Iron determination

Iron mass fraction in the complex was determined by microwave digestion method (*23*). Standard curve was prepared using iron(II) sulphate heptahydrate salt (Sigma-Aldrich, Merck). Sample (100 mg) was weighed in a silica crucible and ashed at 550 °C for 8 h. A volume of 10 mL solution of triple acids (nitric acid/perchloroacetic acid/sulfuric acid in a ratio 3:2:1; Thermo Fisher Scientific) was added to ash and heated on the hot plate for complete dissolution. It was then diluted 100 times with triple distilled water. Finally, iron was determined using atomic absorption spectroscope (AA-7000; Shimadzu, Tokyo, Japan) at *λ*_max_=248.3 nm.

### Colour estimation

Hunter colourimeter Colorflex® (Hunter Associates Laboratory Inc., Reston, VA, USA) was used to quantify the extent of change in the colour of spray-dried WPC–Fe complex in comparison to that of the commercial WPC. The instrument was standardised with standard reference tile (tile coordinates: whiteness *L*=50.83 to 93.00, redness to greenness *a*=0.92 to –26.27 and yellowness to blueness *b*=1.70 to 12.12). Powder sample (50 g) was evenly placed on a clean and dry glass beaker for the evaluation of the colour values.

### Free fat content

Free fat content was quantified using solvent extraction method as per Nijdam and Langrish (*24*) with some modifications. A mass of 10 g powder and 100 mL petroleum ether were placed to a 250-mL flask and shaken for at least 10 times. Then, it was left to stand for 15 min and filtered through Whatman paper no. 42 (Whatman International Ltd., Kent, UK). The liquid was collected in a Mojonnier flask. The process was repeated and the total volume of the filtrate thus collected was evaporated on a heating plate. The dish was then transferred to an oven (Tempo Instruments Pvt. Ltd.) maintained at 100 °C for 1–2 h until constant mass was obtained. The mass was recorded as free fat content.

### Microstructure analysis by SEM

The microstructure of WPC–Fe complex was examined by scanning electron microscope (SEM, model EVO 18; Zeiss, Tokyo, Japan). Spray-dried powder samples were sprayed on aluminium stub-based dual adhesive tape. Samples mounted on the ion coater were coated with gold (20 nm thickness) at 6.66-9.33 Pa for 4 min keeping the ion current at 6 mA. Samples were finally evaluated by SEM under high vacuum (0.012 Pa) at an acceleration voltage of 15 kV and micrographs were captured.

### In vitro bioaccessibility of bound iron from WPC–Fe complex

Bioaccessibility of bound iron from WPC–Fe complex and iron salt was evaluated by dialysis method of Sachdeva *et al.* (*25*). Approximately 5 mL sample solution (WPC–Fe complex and iron salt adjusted to 500 µM iron concentration) were transferred to a flask and 1.92 mL of saliva solution (pH=6.5) were added after which the samples were incubated (Narang Scientific Works Pvt. Ltd, New Delhi, India) in a shaking water bath (SUB Aqua 18 Plus; Grant Instruments) at 37 °C and 95 rpm for 5 min. After incubation, 2.89 mL of gastric juice were added, pH was adjusted to 1.1 with 1 M HCl (Thermo Fisher Scientific) and the solution was incubated at 37 °C for 2 h. On the day of the assay, freshly prepared duodenal juice (5.35 mL) and bile solution (1.92 mL; both Sigma-Aldrich, Merck) were added to the solution after adjusting the pH to 7.8. Final volume of approx. 15 mL of the solution was then incubated at 37 °C for 3 h. Further, it was transferred to an Amicon UF centrifuge tube (Sigma Aldrich, Merck) (MM cut-off 10 KDa) and centrifuged (high-speed refrigerated centrifuge model 6500; KUBOTA Corporation) at 12 000×*g* for 30 min. Iron concentration in the permeate was then determined by atomic absorption spectroscope (AA-7000; Shimadzu) to estimate the digestibility of the added iron under simulated gastrointestinal conditions. Bioaccessibility of iron was calculated from the concentration of the iron that had passed through the ultrafiltration membrane proportional to the total iron concentration of the sample. Bioaccessibility was calculated as follows:

FTB-57-331-e13.eps

where *γ*(Fe) is the concentration of iron (mg/L).

### Statistical analysis

The results are expressed as mean value±standard error of the mean. One-way analysis of variance (ANOVA) followed by Fisher’s least significant difference test served to evaluate the significance. SAS Studio v. 5.1 (*26*) served for data computation.

## RESULTS AND DISCUSSION

### Proximate chemical analysis of WPC and WPC–Fe complex

The proximate composition analysis of WPC and WPC–Fe complex was determined and a significant difference (p<0.05) in protein, fat, lactose and ash content was observed (Table 1). This was due to the fact that the fat fraction separates out as flakes in the supernatant during centrifugation and further during the ultrafiltration process, a large part of the soluble components, especially lactose, is removed as permeate. Therefore, there was a significant reduction (p<0.05) in the fat and lactose content and a consequent increase in the protein and ash content of WPC–Fe complex compared to WPC. There was no significant (p<0.05) difference in the moisture content of WPC and WPC–Fe complex (Table 1).

**Table 1:** Analysis of whey protein concentrate (WPC) and WPC–Fe complex

Component	WPC*	WPC	WPC–Fe complex
*w*(protein)/%	80	(81.7±0.5)^a^	(83.6±0.3)^b^
*w*(lactose)/%	5	(4.6±0.1)^a^	(3.39±0.03)^b^
*w*(fat)/%	8	(7.28±0.04)^a^	(4.5±0.1)^b^
*w*(moisture)/%	-	(4.59±0.07)^a^	(4.72±0.09)^a^
*w*(ash)/%	-	(2.88±0.01)^a^	(3.4±0.1)^b^

Data are presented as mean value±SEM (*N*=3). *Data from the label on the packet (Davisco Foods International Co.). Mean values within rows with different lowercase letters in superscript are significantly different (p<0.05) from each other

### Iron content of WPC–Fe complex

Iron content of the WPC–Fe complex was estimated to be (11.0±0.2) mg/g of the powder (Table 2). Thus, the complex had an adequate iron content and could be used for various food applications as an organic iron fortifier.

**Table 2:** Physicochemical characteristics of whey protein concentrate (WPC) and WPC–Fe complex

Parameter	WPC	WPC–Fe complex
*w*(free fat)/%	(0.79±0.08)^a^	(1.4±0.2)^b^
*ρ*(loose bulk)/(g/mL)	(0.21±0.00)^a^	(0.15±0.00)^b^
*ρ*(tapped bulk)/(g/mL)	(0.37±0.00)^a^	(0.29±0.004)^b^
Carr index/%	(49.7±0.3)^a^	(42.01±0.01)^b^
Hausner ratio	(1.98±0.01)^a^	(1.72±0.00)^b^
L* value	(86.98±0.01)^a^	(78.16±0.02)^b^
a* value	(0.40±0.03)^a^	(5.44±0.01)^b^
b* value	(18.11±0.05)^a^	(22.62±0.02)^b^
*w*(Fe)/(mg/g)	-	(11.0±0.2)^b^
*d*(particle)/nm	(295.5±1.4) ^a^	(248.1±1.7)^b^
ζ-potential/mV	(-12.7±1.1)^a^	(-16.2±1.4)^b^

Data represent mean value±SE of three determinations. Values with different superscripts within the row differ significantly (p<0.05). L*=lightness, a*=redness, b*=yellowness

### Colour value of WPC–Fe complex

Colour of any food product is directly related to its overall acceptability by the consumers. A significant difference (p<0.05) in the determinants of the colour value, *viz*. L* value (86.98±0.01 to 78.16±0.02), a* value (0.40±0.03 to 5.44±0.01) and b* value (18.11±0.05 to 22.62±0.02), between the WPC and WPC–Fe complex (Table 2) was observed, suggesting a significant change (p<0.05) in the colour of WPC after complexation with iron. Thus, the WPC–Fe complex was darker, yellower and redder than WPC. Other factors apart from iron that may have contributed to this change in colour are modifications in particle size, particle and pigment concentration, moisture content, and brown pigment developed as a result of Maillard reactions during heat treatment (*27*, *28*).

### Particle size and zeta potential of WPC–Fe complex

Most of the insoluble proteins were removed by centrifugation and hence the particle size diameter significantly reduced (p<0.05). The average particle diameter of WPC was around 295.5 nm, while of the WPC–Fe complex it was 248.1 nm (Table 2). The ζ-potential assesses the surface charge that arises when any material is placed in a fluid. It is a very good index of the degree of the electrostatic repulsive interaction between particles. Solid-liquid interface characteristics can also affect adhesion, flotation and rheological behaviour (*29*). Hence, the ζ-potential of the WPC–Fe complex produced in this study was analysed. Significant differences (p<0.05) were observable in the ζ-potential of WPC after complex formation with iron (–16.2 mV) compared to the WPC in the absence of iron (control) (–12.7 mV) (Table 2). This increase in ζ-potential could be due to the reduced average particle size diameter of WPC–Fe complex.

### Loose and tapped bulk density of WPC–Fe complex

Loose and tapped bulk density are associated with the powder flowability and storage stability. High bulk density is desirable because it signifies reduced packaging, storage and transport costs per kg of powder (*30*). Loose and tapped bulk density values of the spray-dried WPC–Fe complex were 0.15 and 0.29 g/mL, respectively, and of WPC 0.21 and 0.37 g/mL, respectively (Table 2). A slight decrease in the bulk density of WPC–Fe complex in comparison to that of WPC may be correlated with the decreased size of the powder particles. Our findings are in accordance with the earlier results (*31*, *32*).

### Flow properties of WPC–Fe complex

Carr index (compressibility in %) and Hausner ratio are generally used as the index of flow properties of dry powders. Materials having a Hausner ratio higher than 1.34 are regarded to be cohesive and consequently less free to flow (*32*), while non-free-flowing powders have Carr index greater than 25% (*33*). Low Carr index value was observed for WPC–Fe complex (42.01%) compared to that for the WPC (49.7%) (Table 2). Likewise, Hausner ratio for WPC–Fe complex was observed to be 1.72 in comparison to 1.98 for the WPC (Table 2). High Hausner ratio indicates high cohesion between the particles that results in aggregation and reduced flowability (*34*). The above result demonstrates that both the WPC–Fe complex and WPC had poor flowability.

### Free fat content of WPC–Fe complex

Shelf life of the powder is directly associated with its free fat content, which in turn is directly linked with oxidation. Higher free fat content was observed in WPC–Fe complex (1.4%) than that in WPC (0.79%). This can be due to the greater inlet drying temperature used in the spray dryer, which increases the formation of capillaries and vacuoles, resulting in unprotected fat (*35*) and thus greater free fat content.

### Dissolution behaviour of WPC–Fe complex

**Fig. 1:**
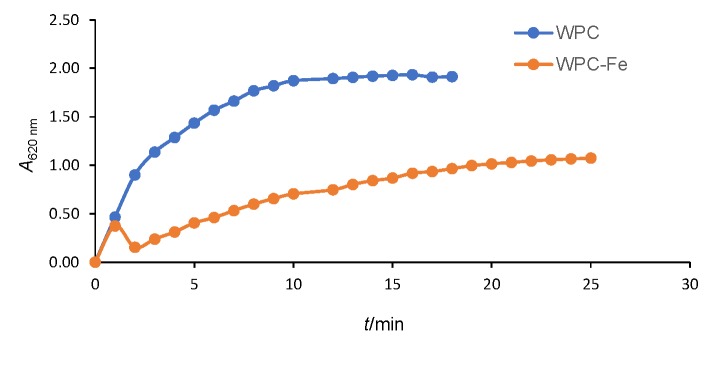
Dissolution behaviour of WPC–Fe complex *vs* whey protein concentrate (WPC) in water at room temperature (30–35 °C)

Powder dissolution behaviour in water determines both the structure and the sensory quality of the food item to which it is added. From Fig. 1, it is evident that both the WPC–Fe complex and WPC dissolved fully in less than 15 min, showing fairly good dissolution power, although the observed WPC–Fe complex dissolution rate was lower. The observed variations in the physicochemical characteristics of commercial WPC and WPC–Fe complex may be due to the differences in the spray drying methods/conditions used in their manufacturing.

### Stability of bound iron in WPC–Fe complex

**Fig. 2:**
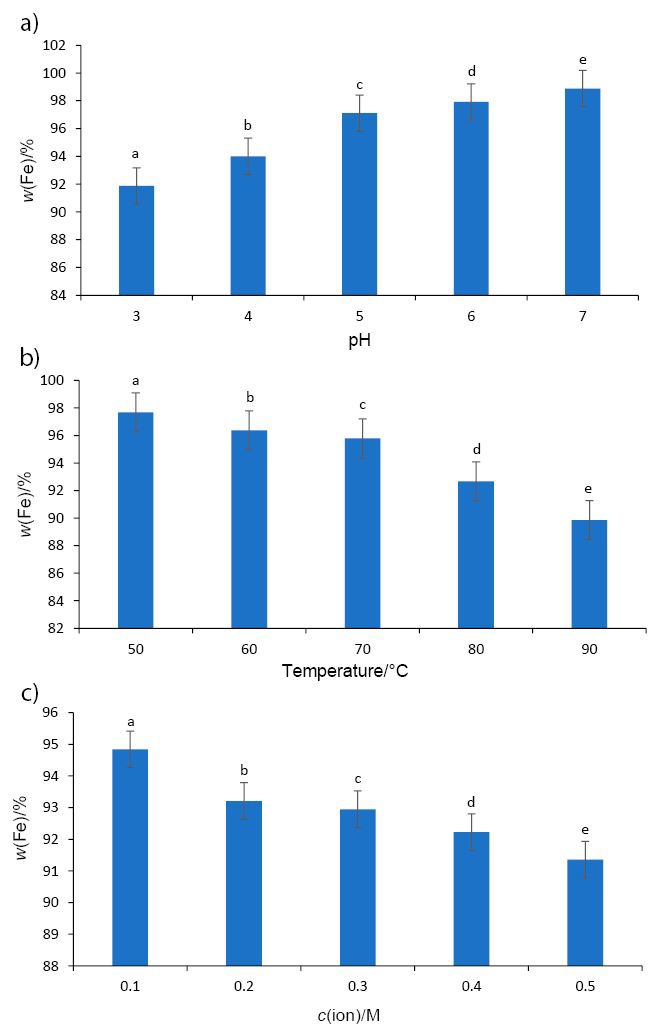
Effect of different processing treatments: a) pH, b) temperature, and c) ion concentration on the retention of the iron in whey protein concentrate-iron (WPC–Fe) complex. Samples with different lowercase letters are significantly different (p<0.05) from each other

Fig. 2 shows the effects of different processing treatments on the retention of bound iron from the WPC–Fe complex in the retentate after ultrafiltration. Control represented the retention of iron in WPC–Fe complex solution subjected to normal conditions, *i.e.* pH~5.75, room temperature ~30 °C, without the addition of NaCl. Significant increase (p<0.05) in iron levels of the retentate of WPC–Fe complex solution was observed when exposed to varying pH (3, 4, 5, 6 and 7), indicating greater stability at higher pH levels. Under these pH conditions, 91.87 to 98.89% of the original iron remained bound to the proteins (Fig. 2a). Heat treatment (50, 60, 70, 80 and 90 °C for 30 min) of WPC–Fe complex solution resulted in significant increase (p<0.05) of free iron in the permeate, which in turn lowered its stability. Nevertheless, even after heat treatment at 90 °C for 30 min, 89.85% of the original iron was still bound to the proteins (Fig. 2b). The increase in ion concentration of WPC–Fe complex solution also resulted in significant rise (p<0.05) of free iron in the permeate, thus lowering the stability (Fig. 2c). However, even at 0.5 M, 91.36% of the original iron was still bound to the WPC. Findings from this study obviously show that the iron retained in the complex was not greatly influenced by various processing conditions, indicating its stability.

### SEM micrographs of WPC-Fe complex

**Fig. 3:**
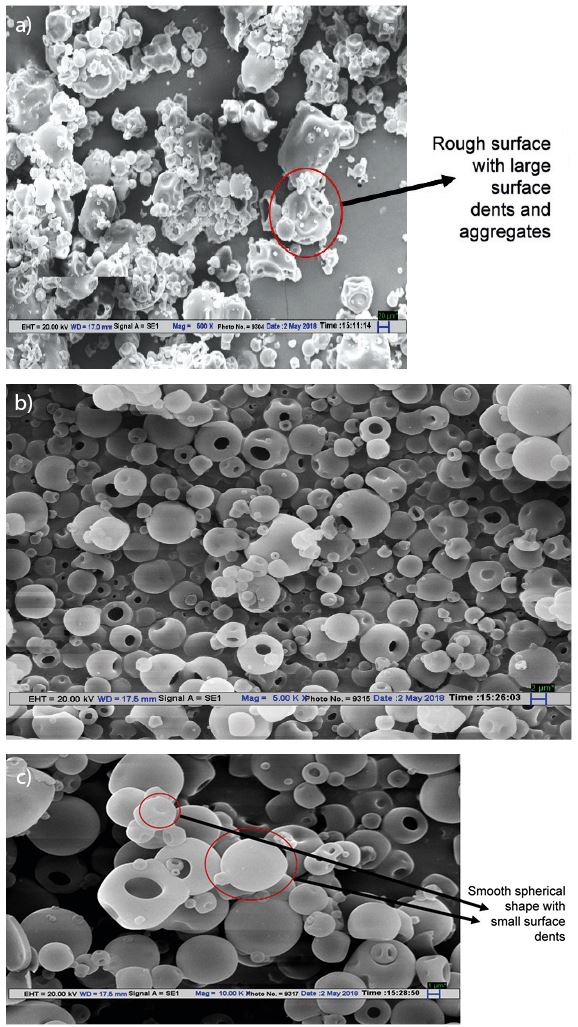
Scanning electron microscopy (SEM) image of: a) whey protein concentrate (WPC) at 500× magnification, b) WPC–Fe complex at 5000× and c) 10 000× magnification

SEM micrographs in Fig. 3 show the surfaces of the WPC and WPC–Fe complex. The WPC had spherical shape with large surface dents. Nijdam and Langrish (*24*) indicated that the formation of big dents on the surface was associated with irregular droplet shrinkage during the early drying phase (Fig. 3a). These dents adversely influence the powder flowability and reconstitution properties (*36*). The spray-dried WPC–Fe complex, on the other hand, showed uniform spherical shape with smooth surface and tiny surface dents (Figs. 3b and 3c).

### In vitro bioaccessibility of bound iron from WPC–Fe complex

**Fig. 4:**
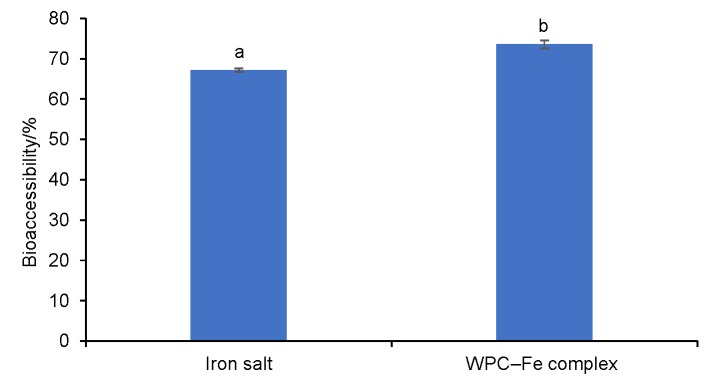
*In vitro* bioaccessibility of iron from WPC–Fe complex in comparison to that of iron salt. Samples with different letters (a and b) are significantly different (p<0.05) from each other

*In vitro* bioaccessibility of iron from the WPC–Fe complex was assessed under simulated gastrointestinal digestion conditions. To ensure the sufficient amount of dialyzable iron in the permeate after ultrafiltration, we used iron concentration of 500 instead of 50 µM in Caco-2 cell monolayer system, according to Hernández-Ledesma *et al.* (*37*). Iron in its free form showed considerably lower digestibility (p<0.05) (67.14%) than the WPC–Fe iron complex (73.52%) (Fig. 4). Reduced digestibility of iron in free form may be due to its insolubility at intestinal pH=7-8. Conrad and Umbreit (*38*) also observed that non-haeme sources of iron (Fe(II) or Fe(III) form) were not available for absorption in the duodenum due to their low bioavailability at intestinal pH. Improved digestibility of iron in the form of WPC–Fe complex may be due to its improved solubility at intestinal pH.

Baech *et al.* (*39*) found that, among different food components, proteins improved iron bioavailability and there are documents (*40*, *41*) on the potential effect of proteins on iron bioavailability. Glahn *et al.* (*42*) noted that peptides produced during protein digestion bind iron to make complexes and increase their intestinal solubility. Mulvihill *et al.* (*43*) indicated that enhanced iron bioavailability in the presence of proteins may be due to the capacity of amino acid sulphydryl groups such as cysteine to reduce Fe(III) to Fe(II). Cremonesi and Caramazza (*44*) interpreted that Fe(II) possesses improved bioavailability owing to the reduced tendency to generate Fe(II) hydroxide precipitates. Proteins have a high content of electronegative residues (carboxylic groups), which are precipitated at pH=2-4 (gastric conditions) keeping the iron in bound form. This could prevent free ion formation and thus transfer the iron to ligands at neutral and alkaline intestinal pH. This mechanism was siutable for succinylated proteins due to the presence of additional carboxylic groups in them. May *et al.* (*45*) also found that iron complexing with organic ligands such as amino acids, carbohydrates, proteins, *etc*. could improve iron solubility by avoiding precipitation at intestinal pH. Nakano *et al.* (*4*) also found that iron-fortified WPC showed better bioavailability than haeme iron in both *in vitro* and *in vivo* experiments because of its better solubility in the small intestine. Shilpashree *et al.* (*46*) also reported that bioavailability of iron from the lyophilised WPC–Fe complex was better than from Fe(II) sulphate. Banjare *et al.* (*8*) reported that milk fortified with spray-dried WPC–Fe complex improved the bioavailability of iron under *in vitro* conditions by preventing the FeSO_4_ from precipitation and increasing its solubility under simulated intestinal conditions. Gandhi *et al.* (*12*) also reported that spray-dried WPC-Fe complex supplementation enhanced the bioavailability of iron in normal weaning and anaemic conditions.

## CONCLUSIONS

Here we developed a method for preparing a whey protein concentrate-iron (WPC–Fe) complex in powder form using spray drying method. Fat and lactose content decreased significantly (p<0.05) in the WPC–Fe complex with consequent increase in protein and ash content compared to WPC alone. The particle size of WPC–Fe complex was smaller than that of WPC, whereas the ζ-potential increased, suggesting the particle stability in any fluid. The flow characteristics and dissolution behaviour of WPC–Fe complex were similar to WPC. The complex had better stability under varying processing conditions. The synthesised WPC–Fe complex was rich in iron and can be used in foods with better iron bioaccessibility. It can be consumed by all age groups and segments of society, thus alleviating the prevalent iron deficiency.
